# Assessment of the impacts of cartap hydrochloride on silver barb (*Barbodes gonionotus*, Bleeker, 1849*)* in rice-fish fields in the Mekong Delta, Vietnam

**DOI:** 10.1007/s10646-025-03014-3

**Published:** 2026-01-03

**Authors:** Nguyen Thanh Tam, Håkan Berg, Nguyen Ngoc Loi, Chau Thi Da, Nguyen Van Cong

**Affiliations:** 1 Faculty of Fishery, Nong Lam University, Ho Chi Minh City, 70000 Vietnam; 2 Department of Physical Geography, Stockholm University, SE-106 91 Stockholm, Sweden; 3 Faculty of Applied Sciences, Ton Duc Thang University, Ho Chi Minh City, 70000 Vietnam; 4 College of Environment and Natural Resources, Can Tho University, 3/2 Street, Can Tho city, Vietnam

**Keywords:** Silver barb, Cartap hydrochloride, Hematoxicity, Acetylcholinesterase, Mekong delta

## Abstract

This study investigates how fish are affected by the insecticide Padan 95SP (95% cartap hydrochloride, CH) used by rice farmers in the Mekong Delta, Vietnam. The impact on survival rate, acetylcholinesterase (AChE), total red (RBC) and white blood cells (WBC), blood glucose and lysozyme activity were measured in silver barb (*Barbodes gonionotus*), raised in a rice field. The field experiment included a control and two treatments, 0.7 kg (R) and 1.4 kg (2 R), of Padan 95SP/ha. Each treatment had three replicates. The water concentration of Padan 95SP decreased from 38.70 and 24.27 to 2.57 and 1.47 µg/L during the first seven days after exposure in the R and 2 R treatments respectively. Although causing only a few fish mortalities, the spraying of Padan 95SP caused significant physiological and immune responses in silver barb. The strongest AChE inhibitions of 18% and 34% were reached after one day in fish from the R and 2 R treatment respectively. The fish recovered fully after seven days. After an initial small increase during the first day, the RBCs, WBCs, and lysozyme activity decreased and were significantly lower in the treatments than in the control between day three and seven, but reached normal levels after 14 days. The glucose levels increased significantly during the first three days but decreased to the same levels as the control after seven days. Despite only few fish mortalities, the results suggests that the use of Padan 95SP has a negative effect on the long-term production of healthy fish in the Mekong Delta.

## Introduction

The Mekong Delta is one of Southeast Asia’s most vital agricultural regions, often referred to as the “Rice Bowl” of Vietnam (Renaud and Kuenzer 2012). Fertile soils, abundant water resources, and favorable climate make it ideal for cultivating a wide range of crops, including rice, vegetables, fruits, fish and shrimps (Berg et al. 2023). Over half of Vietnam’s rice output and a significant portion of its aquaculture production are produced in the Delta (Hui et al. 2022). Agriculture in this region supports the livelihoods of millions of people, contributing to local food security and the national economy (Park, Loc and Tran 2024). However, the agricultural intensification in the Mekong Delta has led to increased use of chemical pesticides (Berg et al. 2023). While these chemicals help control pests and boost crop yields, their widespread application has resulted in unintended consequences for the environment, particularly aquatic ecosystems (Berg and Tam 2018) The runoff of pesticides into rivers, canals, and rice fields has negatively impacted on fish and other aquatic organisms, disrupting biodiversity and threatening the long-term health of aquatic ecosystems (Majumder 2024; Park, Loc and Tran 2024; Renaud and Kuenzer 2012). Wild and farmed fish living in these environments are exposed to multiple pesticides, leading to negative effects on growth rates, reproductive performance, and immune suppression and increased mortalities (Boyd and McNevin 2015; Islam et al. 2022).

According to MARD (2020) the list of pesticides allowed for use in Vietnam includes 4069 commercial products containing 1648 active ingredients. About 32% of the insecticides belong to carbamates (CM). One of these is the active ingredient cartap hydrochloride (CH), a broad-spectrum carbamate insecticide targeting pests such as leaf folder caterpillars, thrips, stem borers, brown planthoppers and armyworms, and it is often detected in surface water in the Mekong Delta (Toan et al. 2013).

Pesticide residues in aquatic ecosystems have been shown to disrupt physiological processes in fish. These disruptions can lead to reduced health, survival rate, growth performance, and increased disease susceptibility (Islam et al. 2022; Mustafa, Al-Rudainy and Salman 2024). Many of these physiological effects occur through impacts on the immune system, making fish more vulnerable to pathogens such as viruses, bacteria, protists, and metazoans (Yang, Lim and Song 2021). Biomarkers, such as hematological parameters and stress markers, have been used to assess fish health under environmentally stressful conditions (Majumder 2025; Wendelaar Bonga 1997). In this context, measurements of red blood cell (RBC) and white blood cell (WBC) counts, blood glucose levels, and lysozyme activity provide valuable insights into the physiological and immune responses of fish exposed to pollutants (Harikrishnan, Balasundaram and Heo 2011; van der Oost, Beyer and Vermeulen 2003).

Changes in RBC counts may indicate anemia or hypoxia due to toxin exposure (Yang, Lim and Song 2021). Blood glucose is another important indicator of stress in fish (Barton and Iwama 1991; Majumder and Kaviraj 2017). Elevated blood glucose levels, or hyperglycemia, often indicates activation of the hypothalamic-pituitary-adrenal (HPI) axis in response to environmental stressors (Wendelaar Bonga 1997). Lysozyme, an important antimicrobial protein, acts as the first line of defense in the innate immune system of fish (Bayne and Gerwick 2001). In fish the lysozyme gene is expressed in bone marrow derived cells (Saurabh and Sahoo 2008) and is found in blood and mucusleukocyte-rich tissues, helping to combat pathogenic microorganisms, especially bacteria (Li et al. 2021). Several studies have shown reduced lysozyme activity following pollutant exposure. For instance, lysozyme activity decreased in rainbow trout (*Oncorhynchus mykiss*) exposed to nitrogen compounds (Möck and Peters 2006) and in carp (*Cyprinus carpio*) exposed to the organophosphate insecticide Chlorpyrifos (Siwicki et al. 1990).These findings are supported by the results of Asgharzadeh, Shareghi and Farhadian (2024) and Li et al. (2013). Also changes in WBC counts often reflect impacts on the immune system (Yang et al. 2021)

Although several studies have focused on the effects of many specific pesticides on fish under controlled laboratory conditions, there is limited information on the health of fish exposure to pesticides in the natural environment, especially in rice field ecosystems (Mustafa, Al-Rudainy and Salman 2024). Fish living in rice fields are under high risks for negative effects of pesticides as a large proportion of the applied pesticides can reach the aquatic environment (Stadlinger et al. 2018). Field assessment of fish health using sublethal indicators, including RBC and WBC counts, blood glucose and lysozyme activity, could substantially improve our understanding of the actual effects of pesticides on wild and farmed fish populations. However, such monitoring is currently lacking in the Mekong Delta. There is therefore an urgent need to identify relevant fish species of ecological and economic importance and to select appropriate physiological indicators for better understanding the sublethal effects of pesticides under field conditions. This would provide a solid scientific foundation for evaluating how agricultural development may impact the Delta’s aquatic resources and guide efforts to minimize these impacts. In this study we focus on Silver barb (*Barbodes gonionotus*), which is widely distributed in freshwater environments in southern Vietnam and is particularly abundant in rice field ecosystems (Rainboth 1996). This species has high economic value thanks to its sweet, soft and fatty meat, and is considered a specialty in the Mekong Delta (Dang 2024; Edwards 2002; Viet 2015). Silver barb is notable for its good adaptability to adverse environmental conditions and rapid growth rate, making this species a priority choice in rice-fish farming systems in the Mekong Delta (Anh et al. 2003; Edwards, Little and Yakupitiyage 1997; Klemick and Lichtenberg 2008).

This study represents the first field-based assessment of the effects of the carbamate insecticide cartap hydrochloride on the health and immune response of silver barb (*Barbodes gonionotus*) in rice field ecosystems of the Mekong Delta. Specifically, it evaluates the impact on acetylcholinesterase (AChE) enzyme activity, survival rate, and immune parameters, including RBC and WBC counts, blood glucose levels, and lysozyme activity. By examining these sublethal indicators, the study aims to fill a critical gap in understanding the effects of pesticide exposure on both wild and farmed fish under natural field conditions. The results of this research provide important data to support the development of early warning systems and strategies for mitigating the ecological and environmental risks posed by pesticide use. Additionally, the findings could contribute to the advancement of more sustainable and eco-friendly agricultural practices in the Mekong Delta, underscoring the importance of safer pesticide applications. Ultimately, this study will help promote the long-term sustainability of both rice and fish production in the region, ensuring healthier and more resilient agricultural systems.

## Materials and methods

### Test animals

Silver barb (*Barbodes gonionotus*) fingerlings (1.51 ± 0.03 g/fish and 2.67 ± 0.14 cm/fish) were bought from a hatchery in Cai Rang district, Can Tho, and transported to a wet laboratory at the College of Environment and Natural Resources, Can Tho University. Upon arrival, they were acclimated for 45 days in 600-liter fiberglass tanks filled with dechlorinated tap water. The stocking density was 100 fish/tank. Water quality during acclimation was maintained within the ranges of pH 7.5–8.0, temperature 30.1–31.6 °C, and dissolved oxygen 4.13–5.93 mg/L. During this acclimation period, the fish were fed twice daily with commercial floating pellets (35% protein, 6% lipid, 14% ash, 7% fibre, 11% moisture) produced by Greenfeed Vietnam Corporation. Additionally, 50% of the water was replaced every 2-3 days to remove faeces and uneaten food. After a 45-day acclimation period in the wet laboratory, the fingerlings were transferred to the study site and further conditioned for five days in a hapa (4 × 10 × 1.5 m) placed within an earthen pond before the experiment setup. Feeding was suspended one day prior to transportation and experimental setup. All experimental procedures involving fish were conducted in accordance with institutional guidelines for the care and use of animals in research. Ethical approval for the study was granted by the Animal Ethics Committees in Nong Lam University ((AEC—NLU) NLU-240618).

### Insecticides

The insecticide used, Padan 95SP (95% cartap hydrochloride), was manufactured by the limited liability company Sumitomo Chemical Co., Ltd. Padan 95SP is used against insect pests such as leaf folder caterpillars, thrips, stem borers, brown planthoppers, and armyworms. The recommended dosage is to spray rice fields with 0.5 – 0.7 Kg/ha.

### Experimental designs

A field experiment was conducted in a rice field to determine the impacts of Padan 95SP insecticide on red and white blood cell counts and blood glucose levels, lysozyme activity and acetylcholinesterase (AChE) activity in silver barb fingerlings. The experiment included a control group and two treatment groups, in which Padan 95SP was sprayed at concentrations of 0.7 kg/ha (R) and 1.4 kg/ha (2 R). The R treatment corresponds to the maximum dose recommended by the manufacturer, while the 2 R treatment represents twice this recommended level. The inclusion of 2 R was not only based on its frequent application by farmers in the Mekong Delta, but also to evaluate the potential agronomic and ecological consequences of such overuse relative to the recommended dose. Each treatment was conducted with three replicates.

**Fig. 1:**
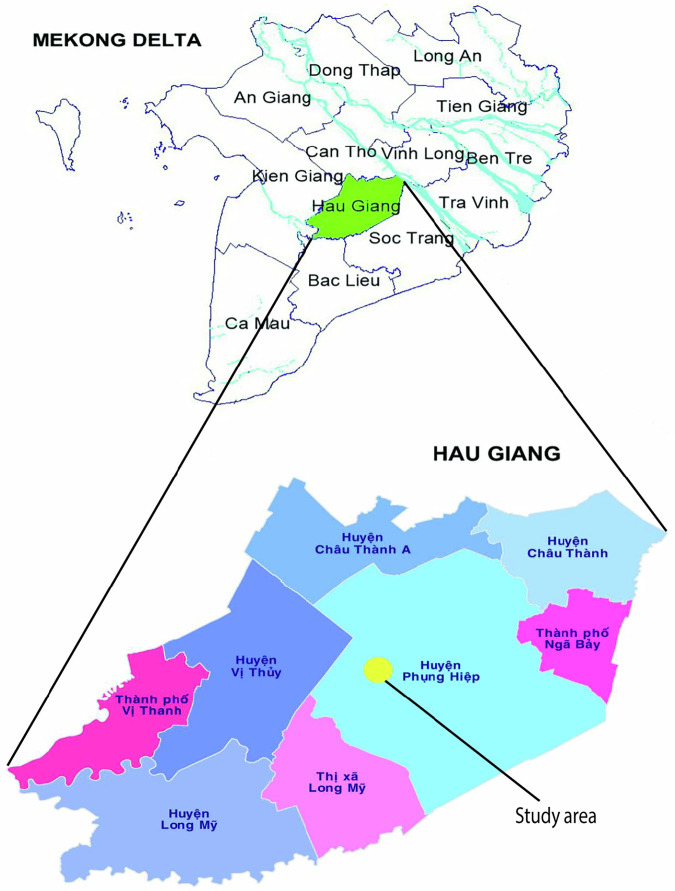
The study was conducted in Phung Hiep districts in Hau Giang, a major rice-fish producing province in the Mekong Delta

The experiment was conducted in nine rice fields in the Phung Hiep district, Hau Giang province, Vietnam (Fig. 1). Each rice field had an approximately area of 1000 m^2^. The rice fields were separated by earthen dikes (25 cm high and 35 cm wide) reinforced with nylon sheets to prevent water exchange between the experimental fields and the surrounding environment. The same local rice variety was used across all experimental plots, sown at a density of 20 kg/1000 m^2^. The rice was harvested 100 days after seeding. Uniform agronomic practices were applied to all plots and the water level was monitored weekly at 8:00 am as well as after heavy rainfall events throughout the experiment.

Thirty-eight days after sowing, three designed areas (1.2 × 2.2 m^2^) along the central axis of each rice field, spaced approximately 15 m apart, were cleared of rice plants. In each cleared area, a nylon cage (1 × 1 × 2 m) was installed. The experimental cages were equipped with covers to prevent the escape of fish and to exclude the entry of potential predators or other harmful organisms. The water depth across all sites was maintained at approximately 20 cm. The cages were left for one day to allow suspended solids to settle from the water. A total of 60 fingerlings (2.39 ± 0.05 g/fish and 4.43 ± 0.36 cm/fish) were randomly selected from the hapa used for acclimation and released into each experimental cage. The fish were fed twice daily with commercial floating pellets containing 35% protein at approximately 5% of their body weight.

Seven days after the fingerlings were released into the cages, corresponding to 45 days post-seeding, insecticides were supplied to farmers, who prepared the spray solutions with water taken directly from the rice fields. The mixtures were prepared to meet the designated treatment concentrations and followed the same procedures that farmers routinely use when applying pesticides in their fields. This practice was considered sufficient to ensure solution homogeneity under typical field conditions. No supplementary mechanical mixing or artificial rainfall were introduced.

Throughout the experiment, water temperature and dissolved oxygen (DO) levels were recorded in the morning (7:00–8:00 AM) and afternoon (14:00–15:00 PM) every second day, using an Orion 830 A DO meter (Thermo, Beverly, MA, USA), whereas pH was measured only in the morning using a Portamess 911 pH meter (Knick, Berlin, Germany).

### Collection of water samples

Water samples were taken from each cage at several intervals throughout the experiment to measure cartap hydrochloride concentrations. The water samples were taken immediately after introduction of the fish, on day 7 (one-hour post-spraying) and thereafter at 1, 3, 5,7 and 14 days after spraying. For each water sample, 1 L of mixed water was collected in brown glass bottles. These samples were kept on ice during transportation to the laboratory, where they were stored at −20 °C until analysis within a week. Pesticide quantification was performed at Hai Dang Chromatography Science Service Joint Stock Company (EDC-HD), Vietnam, following the spectrophotometric method described by Raza (2013) and Parfitt (2000). Water samples were extracted and analyzed under the laboratory’s standard operating procedures, with calibration performed using certified reference standards of cartap hydrochloride. The method achieved a limit of detection (LOD) of 1 µg/L. Method validation included spiking experiments in both distilled and field water, which yielded recovery rates ranging from 96–99%.

### Collection of blood samples

Fish samples were collected at the same time intervals as the water samples. Fish size (weight and length) was measured prior to blood sampling and was homogeneous across replicates. At each sampling time, three fishes were removed randomly from each of the three replicate cages/treatments, resulting in a total of nine fish per treatment and sampling time. Blood was taken from the caudal peduncle with a sterile plastic syringe (2.5 mL) after the fish had been anesthetized using AQUI-S anesthetic (100 ml containing 50% isoeugenol, manufactured by Elanco company, Vietnam) at a dose of 25 ppm. A blood sample from each fish was analyzed separately rather than pooled. This approach was chosen to preserve inter-individual variation and to provide sufficient replication for statistical analyses, as pooling would have reduced the number of independent samples and potentially masked biologically relevant differences among individuals. Accordingly, values reported as “mean of 27 samples” represent the average of 3 blood samples × 3 cages × 3 rice fields. The blood samples were transferred into two sterile vials: i) the first vial contained 1.26 mg of Ethylenediaminetetraacetic acid (EDTA) anticoagulant for hematological analysis. The second vial, without anticoagulant, was used to separate the serum. The sample was centrifuged for 10 minutes at 3000 rpm, then the serum was separated and stored at 4°C until biochemical and lysozyme analyses. All samples, along with the fish, were kept on ice and promptly transported to the laboratory, where they were immediately processed to remove the brain for sub-lethal effect measurements including Acetylcholinesterase (AChE) activity, lysozyme, total red and white blood cells and glucose levels. The survival rates were calculated at the end of experiment.

### Cholinesterase assay

Before removal of the fish’s brain, the fish was weighed individually using an electronic balance (BP410S; Sartorius, Goettingen, Germany), and its total length was measured including the tail fin. The brain was then carefully dissected out on ice and immediately placed in a pre-weighed Eppendorf tube kept on ice before measuring its weight. Each brain was homogenized on ice in 6 ml of 0.1 M phosphate buffer (pH 7.4, prepared by mixing mono and dibasic sodium hydrogen phosphate) using a glass homogenizer. The resulting homogenates were transferred into 10 ml glass tubes and centrifuged at 2,000 rpm at 4 °C for 20 min (Centrifuge 4k15; Sigma, Osterode am Harz, Germany). Then, 1.5 ml of the supernatant was collected and transferred to an Eppendorf tube, and kept on ice for AChE analyses within 12 h. The ChE activity was determined according to the method described by Ellman et al. (1961). All measurements were performed in an air-conditioned room at 25 °C. For each measurement, a cuvette was prepared containing 2.65 ml of the 0.1 M phosphate buffer (pH 7.4), 100 µl of 3 mM 5,5 dithio-bis (2-nitrobenzoic acid) (DTNB; Sigma Aldrich Chemie, Steinheim, Germany). Immediately before measurement, 50 µl of 10 mM acetylthiocholine iodide (Sigma Aldrich Chemie) and 200 µl of the supernatant was added, and the solution was mixed well. Blanks were prepared with 200 µl of buffer instead of the supernatant. Two blanks were used for each sample measurement. Enzyme ChE activity was detected using an ultraviolet/visible spectrophotometer (model UV2 2000E; ATI Unicam, Cambridge, UK) for 10 cycles (3 min and 18 s) with auto interval (22 s) at a wavelength of 412 nm, during which time the increase in absorbance with time was linear. The results of these measurements were expressed as a rate (absorbance per min) and the ChE activity calculated.

### Total red and white blood cell counts, Glucose analyses and Lysozyme assays

Approximately 20 μL of blood from the EDTA vial was used for determination of red and white blood cell counts using an automated blood cell analyzer (LUNA-II™ Automated Cell Counter, Logos Biosystems Co., Ltd., Korea)

Plasma glucose levels were measured in 2–10 µL blood samples with Elite Blood Glucose Test Strips (Glucometer Elite, Bayer Corporation, USA).

The serum separated from the second sterile vial was used to determine the lysozyme activity of the silver barb serum via the turbidity assay method by Stolen (1995).

### Data analyses

Data was checked for normality and variance homogeneity prior to statistical analysis. The Chi-squared test was applied for data that did not meet normality and variance homogeneity requirement. Data was analyzed with one-way ANOVA and Dunnett’s post-hoc test for multiple comparisons. SPSS for windows (Ver 17.0; SPSS, Chicago, IL, USA) was used to analyze the data.

## Results and discussion

### Water parameters

Water temperature is an important factor affecting the growth and development of most aquatic organisms, and the mean temperature in the water across the experimental fields ranged from 28.0 ± 0.4 to 30.2 ± 0.2 °C (Table 1). There was no statistically significant difference in temperature among fields throughout the experiment. Silver barb thrive in water bodies with temperatures ranging from 22–30 °C, with an optimal range of 24–28 °C (Gojendro et al. 2024; Mazumder et al. 2024). Thus, the temperature in experimental fields was within the suitable range for normal growth and did not cause adverse effects on the experimental fish.

**Table 1:** Water parameters throughout the field experiment

Water parameters	Control	R^a^	2R^b^
Temperature (^o^C)	28.3 ± 0.2–30.1 ± 0.3	28.2 ± 0.3–30.2 ± 0.2	28.0 ± 0.4–30.2 ± 0.1
DO (mg/L)	1.3 ± 0.2–1.9 ± 0.2	1.3 ± 0.3–1.9 ± 0.4	1.3 ± 0.5–1.9 ± 0.2
pH	6.6 ± 0.2–6.7 ± 0.1	6.6 ± 0.1–6.7 ± 0.2	6.6 ± 0.3–6.7 ± 0.2

Data are expressed as means ± SE

^a^R= the highest recommended dose by the manufacturer (0.7 Kg/ha)

^b^2R= the dose commonly used by farmers (1.4 Kg/ha)

The water pH values in the experimental fields ranged between 6.6 ± 0.1 and 6.7 ± 0.2 and remained relatively stable across measurement and treatments throughout the study (Table 1). Gojendro et al. (2024) stated that the optimal pH range for silver barb is between 7 and 8, but the fish can live in slightly acidic waters with a pH as low as 5.5. This indicates that the pH conditions in the experiment were still within an acceptable range for the normal growth and development of the fish, albeit not at an optimal level.

The mean dissolved oxygen concentrations in the water in all rice fields ranged from 1.3 ± 0.2 to 1.9 ± 0.4 mg/L (Table 1). There were no statistically significant differences in DO levels among the rice fields at any time. The DO levels were relatively low compared to the normal oxygen requirements by many fish species. However, Gojendro et al. (2024) reported that silver barb can tolerate DO levels as low as 0.656 mg/L, so the low DO levels in the experiment probably did not affect normal growth and development of the fish, although an increased stress on the fish from these low level not can be excluded. However, the fact that there was no significant difference in any of these water parameters, indicates that the observed effects on fish primarily were caused by CH.

### Water concentrations of CH

**Fig. 2:**
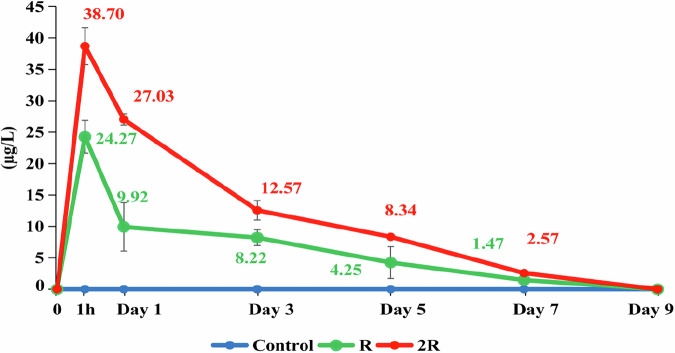
Concentrations of CH in water from rice fields sprayed with Padan 95SP at a concentration of 0.7 kg/ha (R) and 1.4 kg /ha (2 R). The vertical bars show standard error. Each data point corresponds to the mean of 9 samples

The concentrations of CH in the water across the treatments throughout the experiment are presented in Fig.  2. The water concentrations of CH increased immediately after spraying and peaked after one hour with the highest levels in 2 R (38.70 µg/L). The CH then started to decline for both treatments, but the levels remained higher in 2 R than in R. The average concentration after five and seven days were 8.34 and 2.57 µg/L (2 R), and 4.25 and 1.47 µg/L (R), respectively. After nine days the levels were below the detection limit for both treatments. Centanni et al. (2024) pointed out that high doses not only increased the initial concentration in water but also prolonged the residual time of the pesticide, leading to a more serious risk to aquatic organisms. The results are consistent with the study of Mitra et al. (2024), who showed that carbamate compounds have a relatively short half-life, usually not exceeding 10 days under tropical environmental conditions. In addition, Tomašević et al. (2019) stated that chemical, photochemical, and biological degradation mechanisms cause a rapid decline in the concentration of carbamate compounds such as cartap hydrochloride in aquatic environments. The environmental conditions in the experimental rice fields, including high temperatures (28.2–30.2 °C) and strong light, provided suitable conditions for such degradation processes, contributing to the rapid reduction of Padan 95SP concentrations in the water. In addition, biological factors also play an important role in these degradation processes. According to Tien et al. (2013) and Chapalamadugu and Chaudhry (1992), microorganisms in water are capable of converting carbamate and organophosphates based compounds into less toxic degradation products, thereby contributing to the rapid reduction of pesticide toxicity in the aquatic environment.

### Survival rate

**Fig. 3:**
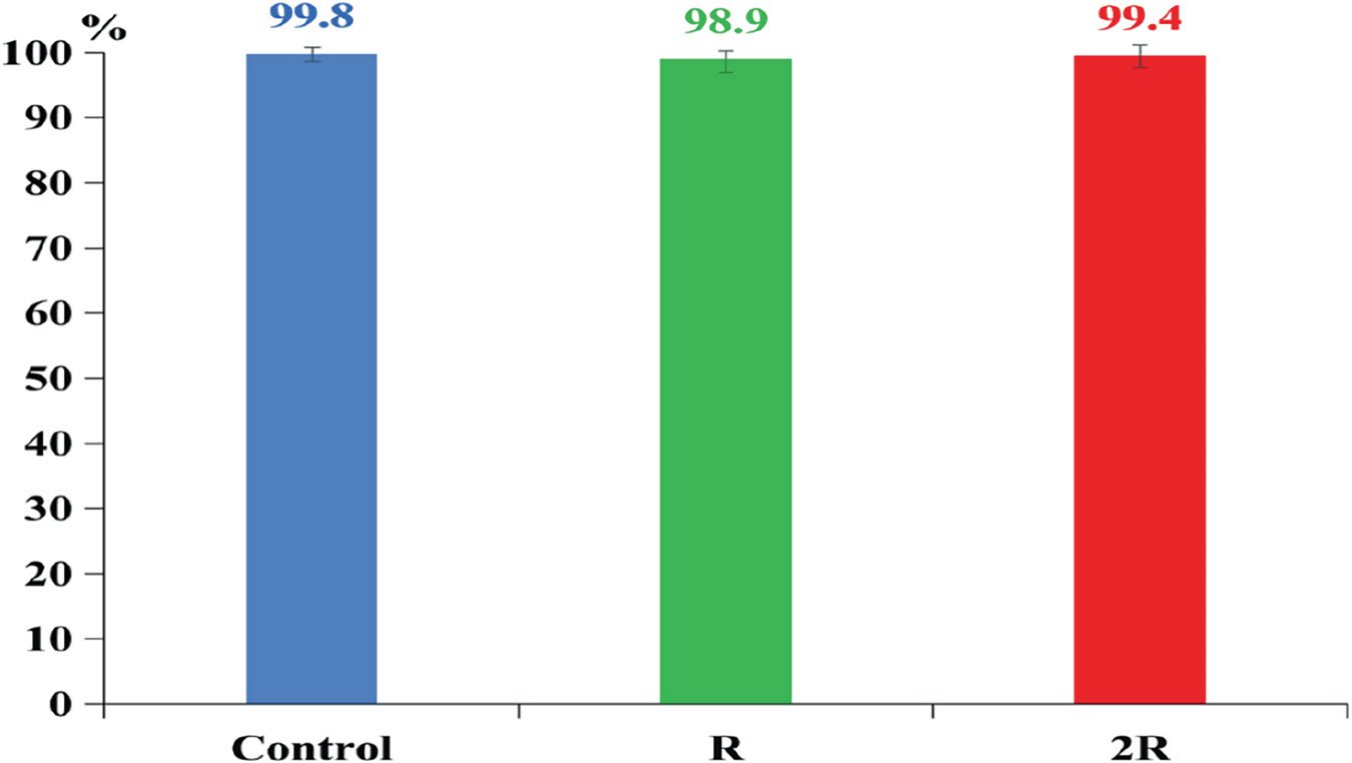
Effect of CH on survival rate of silver barb. C: Control; R: the highest recommended dose by the manufacturer (0.7 kg Padan 95SP/ha); 2 R: the actual dose used by many farmers (1.4 kg Padan 95SP/ha). The vertical bars show standard error. There was no significant difference between means at the same sampling times (one-way ANOVA, *P* < 0.05). Each data point corresponds to the mean of 9 samples

The average survival rates in the control, R and 2 R treatments were 99.8, 98.9 and 99.4%, respectively (Fig. 3), with no statistically significant differences among treatments (*P* > 0.05). Thus, the highest dose of CH recommended by the manufacturer and the dose commonly used by farmers (usually double the recommended dose) did not affect the survival rate of silver barb cultured in rice fields.

### Brain AChE inhibition in silver barb

**Fig. 4:**
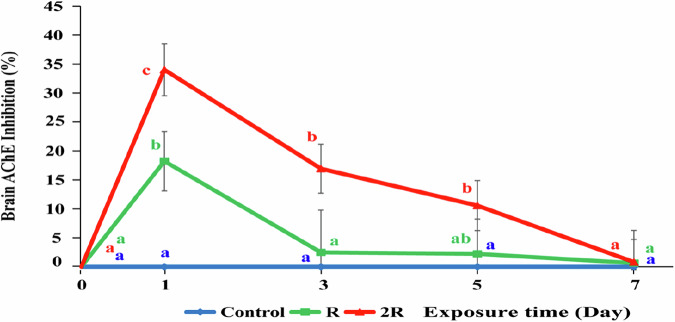
Brain acetylcholinesterase (AChE) inhibition (%) in silver barb fingerlings before and after spraying rice fields with Padan 95SP at a concentration of 0.7 kg/ha (R) and 1.4 kg /ha (2 R). The vertical bars show standard error. Different letters show that the data are significantly different between means at the same sampling times (one-way ANOVA, *P* < 0.05). Each data point corresponds to the mean of 9 samples

The AChE inhibition levels in silver barb fingerling across the treatments throughout the experiment are presented in Fig. 4. Padan 95SP had a significant impact on the AChE activity of silver barb and the AChE inhibition level in fish was proportional to the exposure concentration. Before exposure to CH, the average AChE activity in fish from the control and both treatments ranged from 8.25 to 8.38 µM/g/min, with no statistically significant differences between the treatments and the control (*P* > 0.05).

The highest brain inhibitions occurred one day after exposure and were 34.0 and 18.0% for 2 R and R respectively, which were significantly different compared to the control (*P* < 0.05). According to Sogorb and Vilanova (2002), active ingredients belonging to the carbamate group have the ability to inhibit the enzyme AChE through a mechanism of binding to the catalytic center, reducing the activity of the enzyme.

At day three the AChE inhibition in fish from R was 2.2%, showing an almost complete recovery, with similar activity as in fish from the control (*P* > 0.05). The AChE inhibition in fish from 2 R (16.9%) also showed signs of recovery, but was still statistically higher than in the fish from R and the control (*P* < 0.05). By the fifth day after exposure to CH, the AChE inhibition in fish from 2 R was still 10.6%, which was significantly higher than that of the control (*P* < 0.05).

Seven days after the CH application, 2 R had also recovered fully (*P* > 0.05). The reduced inhibition with time may be due to increased protein synthesis by fish to compensate for AChE enzymes blocked by CH (Ram et al. 2003). According to Assis, Bezerra and Luiz (2011), inhibition of the AChE activity by carbamates such as CH is usually rapid. However, after the exposure has ended the AChE enzyme activity can recover rapidly in affected organisms (Stenersen 2004; Tam et al. 2016). This is explained by the fact that the ester bond between the active carbamate group and the hydroxyl group of AChE is relatively weak, allowing some AChE enzymes to be released from the carbamate bond and resume function without undergoing a new synthesis (Moralev and Rozengart. 2007).

Compared to some other pesticides used in the Mekong Delta, CH has a higher 96-h LC_50_ (0.36 mg/L) to silver barb than Chlorpyrifos ethyl (0.119 mg/L, (Cong, Danh and Nam 2021)) and Carbosulfan (0.275 mg/L (Cong et al. 2021)). However CH is more toxic to silver barb than Fenobucarb and Quinalphos, which have 96-h LC_50_ values of 12.7 mg/L and 0.856 mg/L, respectively (Anh et al. 2012)

### Effects of CH on the immune systems endpoints

#### Total red and white blood cells count

**Fig. 5:**
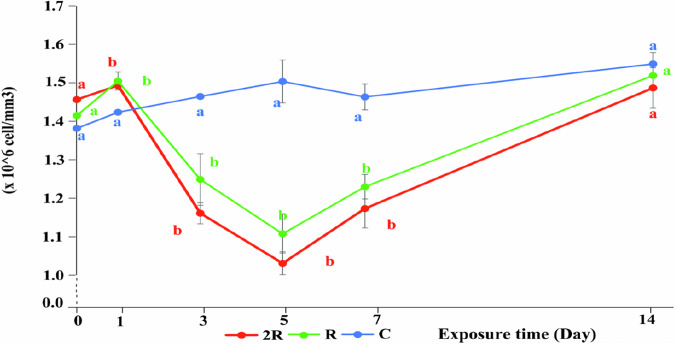
Effect of CH on red blood cell count (×10^6^ cells/mm^3^) in silver barb. C: Control; R: the highest recommended dose by the manufacturer (0.7 kg Padan 95SP/ha); 2 R: the actual dose used by farmers (1.4 kg Padan 95SP/ha). The vertical bars show standard error. Different letters show that the data are significantly different between means at the same sampling times (one-way ANOVA, *P* < 0.05). Each data point corresponds to the mean of 27 samples

**Fig. 6:**
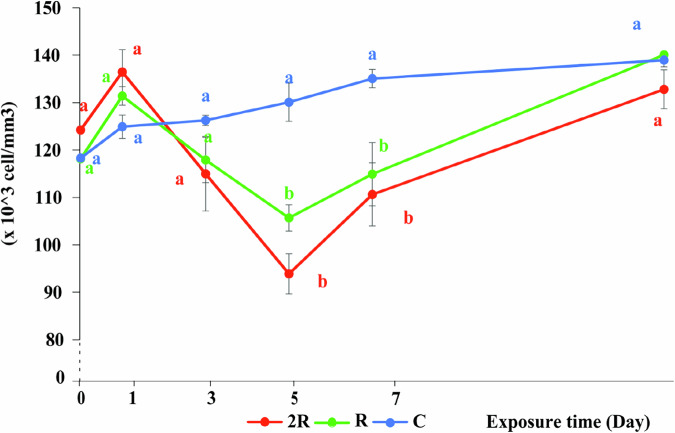
Effect of CH on white blood cell count (×10^3^ cells/mm^3^) in silver barb. C: Control group; R: the highest recommended dose by the manufacturer (0.7kg Padan 95SP/ha); 2R: the actual dose used by farmers (1.4 kg Padan 95SP/ha). The vertical bars show standard error. Different letters show that the data are significantly different between means at the same sampling times (one-way ANOVA, *P* < 0.05). Each data point corresponds to the mean of 27 samples

Both treatments of CH affected the number of RBCs and WBCs in fish significantly compared to the control (Figs. 5, 6), and clearly showed the effects of CH on the hematopoietic function and adaptive mechanism of fish. One day after exposure, the RBC counts were 1.50 (R) and 1.49 (2 R) x10^6^ cells/mm^3^, which was significantly higher than that of the control group (1.42 × 10^6^ cells/mm^3^) (*P* < 0.05) (Fig. 5 ). This increase in RBCs suggests a defense response to acute stress, in which more RBCs are temporarily mobilized from storage reservoirs such as the spleen to increase the oxygen-carrying capacity to meet the high oxygen demand for metabolism under stress (Jern et al. 1989; Paulson, Hariharan and Little 2020).

Five days after the exposure, the RBC counts in R and 2 R had decreased to the lowest levels of 1.11 and 1.03 × 10^6^ cells/mm^3^ respectively (Fig. 5), which was statistically significant different compared to the control (1.50 × 10^6^ cells/mm^3^) (*P* < 0.05) (Fig. 5). The significant decrease in the number of RBC on the third and fifth days may be due to the decrease in the activity of the hematopoietic system. According to Joshi, Harish and Bose (2002), the decrease in the number of RBC is due to the inhibition of erythropoiesis and the acceleration of erythrocyte degeneration in the hematopoietic organs. Low dissolved oxygen conditions in the experimental rice fields may also be the cause of the decrease in RBC, leading to the destruction of RBC or the lack of hemoglobin in the cell medium (Chen et al. 2004). This should also have caused a decrease in the control, which tended to increase. Ullah et al. (2022) showed that after exposure to the pesticide cypermethrin, the number of RBC in grass carp was sharply reduced due to stress and damage to blood cells.

After the fifth day, the RBC count in the CH treatment began to recover, but they were still significantly lower than that of the control at day 7 (*P* < 0.05) and only returned to almost the same level as the control on day 14^th^. The recovery could be explained by the decreased CH concentrations in the water and that the fish defense and elimination mechanisms had helped to restore liver functions, thereby supporting increased red blood cell production. Hossain et al. (2022a) and Zahran et al. (2018) reported a recovery of RBCs in tilapia after the exposure to chlorpyrifos had stopped, which was similar to the recovery in fish exposed to CH in this experiment.

One day after exposure to CH, WBC counts in fish from the two treatments were 131.4 (R) and 136.5 (2 R) x10^3^ cells/mm^3^, which was slightly higher compared to the control group (124.9 × 10^3^ cells/mm^3^). However, this difference was not statistically significant (*P* > 0.05) (Fig. 6). Chen et al. (2004) pointed out that when exposed to stressors, fish activate the immune defense mechanism through an increase in the number of WBC. The increase in WBC count is associated with increased antibody synthesis, which aids in the recovery process and increases the survival of fish exposed to pesticides (Joshi, Harish and Bose 2002).

Similar to the RBCs, the WBC counts in fish from the CH treatments decreased to their lowest levels of 105.7 (R) and 93.9 (2 R) ×10^3^ cells/mm^3^on the fifth day (Fig. 6), which were statistically significantly lower than in the control group (130.1 × 10^3^ cells/mm^3^) (*P* < 0.05). This result is consistent with those of many other studies. Dhabhar (2009) showed that pesticides can stimulate an initial immune response by increasing the number of WBC, however, exposure under longer periods or to high doses often leads to immunosuppression, similar to the trend of WBC in fish found in this study. Yang, Lim and Song (2021) also showed that pesticide induced stress increases leukocyte mortality or disrupts the function of immune cells, resulting in a significant decrease in WBC counts. After day five, WBC counts in the CH exposed fish started to recover, and eventually returned to levels comparable to the control group by day 14 (Fig. 6). This supports the findings by Hossain et al. (2022a), who found that the fish immune system recovered when the exposure to insecticides ended.

#### Blood glucose

**Fig. 7:**
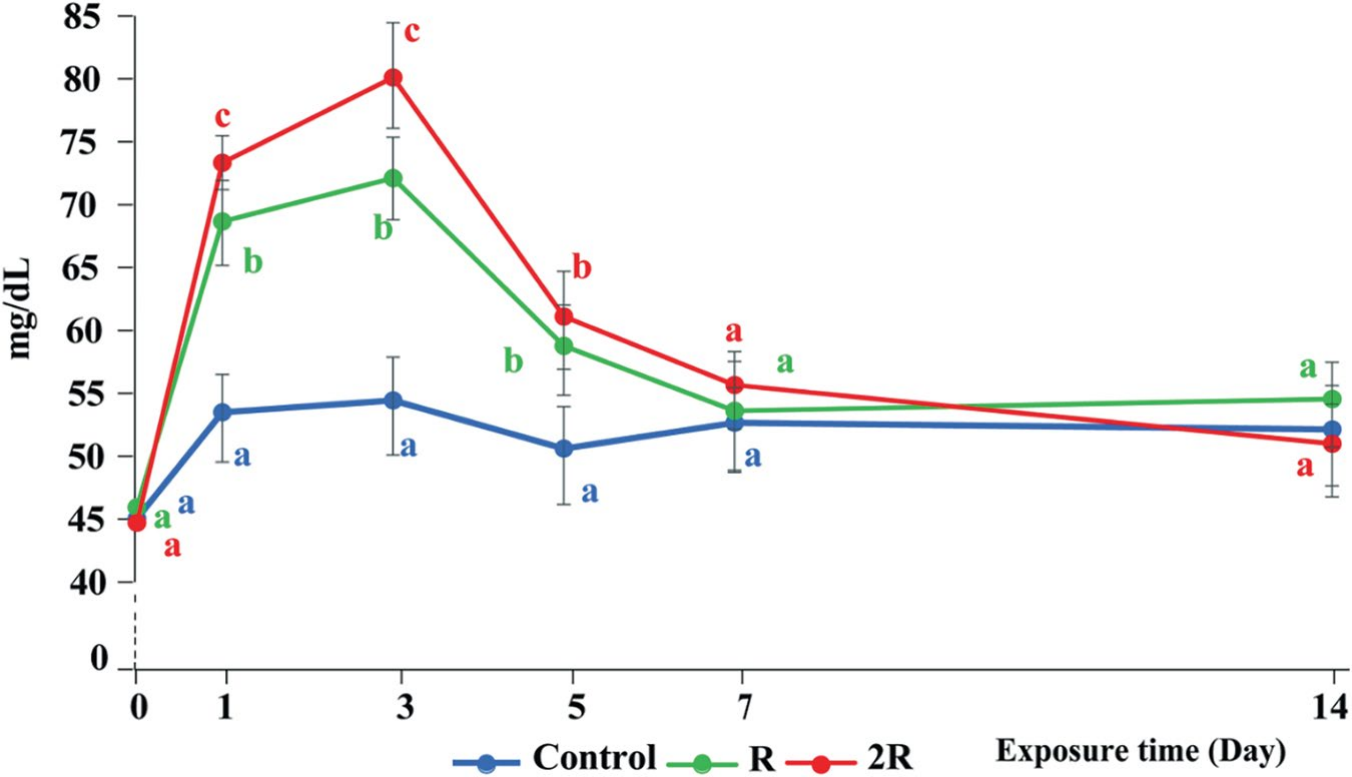
Effect of CH on blood glucose (mg/dL) in silver barb. C: Control; R: the highest recommended dose by the manufacturer (0.7 kg Padan 95SP/ha); 2 R: the actual dose used by farmers (1.4 kg Padan 95SP/ha). The vertical bars show standard error. Different letters show that the data are significantly different between means at the same sampling times (one-way ANOVA, *P* < 0.05). Each data point corresponds to the mean of 27 samples

After exposure to CH, the blood glucose in fish increased rapidly and became statistically significantly higher than the blood glucose in the control fish during the first five days (*P* < 0.05) (Fig. 7 ). In addition, the blood glucose levels in fish from 2 R were significantly higher than that of fish in R, during the three first days after exposure to CH (*P* < 0.05). At days seven and fourteen, the blood glucose levels in fish in all treatments ranged from 51.0 to 55.7 mg/dL and there were no significant differences among treatments and the control (*P* > 0.05). These results are similar to the research on silver barb by Kole et al. (2022) who found that on the first day after exposure to the insecticide Sumithion blood glucose levels in fish increased, followed by a significant decrease on the seventh day. Winkaler et al. (2007) suggested that when exposed to stress fish energy demand increases, leading to increased glucose production and transport from the liver to energy-consuming organs to counteract the harmful effects of the stressors (Hori et al. 2006). The decrease in blood glucose on the seventh day in this study could indicate that the decreased CH levels in the water did not cause any substantial stress to the fish anymore and that the fish had recovered, which also was supported by recovered AChE activity at day 7 (Fig. 4).

#### Lysozyme

**Fig. 8:**
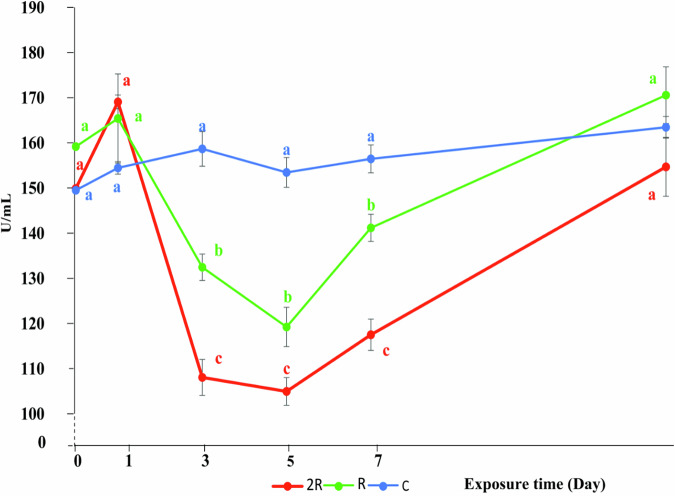
Effect of CH on lysozyme (U/ml) in silver barb. C: Control group; R: the highest recommended dose by the manufacturer (0.7 kg Padan 95SP/ha); 2 R: the actual dose used by farmers (1.4 kg Padan 95SP/ha). The vertical bars show standard error. Different letters show that the data are significantly different between means at the same sampling times (one-way ANOVA, *P* < 0.05). Each data point corresponds to the mean of 27 samples

The lysozyme activity of silver barb in the control group remained quite stable throughout the experiment, with only minor fluctuations ranging between 149.50–160.11 U/mL (Fig. 8). In contrast, the lysozyme activity of fish from the R and 2 R treatments fluctuated much more. The first day after exposure to CH, the lysozyme activity in the R and 2 R treatments was 165.39 and 169.06 U/ml, respectively, which was higher than that of the control group (154.44 U/ml) (Fig. 8), although not statistically significant (*P* > 0.05). This increased lysozyme activity indicated an activated immune system, which is a non-specific immune response when fish are exposed to stressors and plays a role in the initial defense mechanism by the fish to stressors (Biller et al. 2021).

However, after that, the lysozyme activity of fish in the CH treatments decreased rapidly to the lowest level of 119.2 (R) and 105.0 (2 R) U/mL on day five. During day three and seven the lysozyme activity in the fish was statistically significantly different between the treatments and the control (*P* < 0.05). The rapid decrease in lysozyme activity may have been caused by a temporary suppression of the immune system by the direct action of CH, which impairs the ability to produce lysozymes. Previous studies have shown that toxic chemicals, including insecticides, can induce oxidative stress and reduce the activity of immune enzymes in animals (Amenyogbe et al. 2021; Banerjee, Koner and Ray 1996; Gupta 2006; Sule, Condon and Gomes 2022). Thus the decreased lysozyme activity may be related to the effects of insecticides on the immune system or immune cells, leading to a decrease in the body’s ability to defend itself (Khoshnood 2024).

After day five, and similar to the levels of RBCs and WBCs, the lysozyme activity in fish from the CH treatments showed a recovery trend, and finally returned to levels comparable to the control group by day 14 (Fig. 8 ). This recovery demonstrates the ability of silver barb to recover from the negative effects of CH at sublethal concentrations over a long period of time. Studies have shown that although blood parameters and immune enzymes may decrease shortly after exposure to toxins, animals can recover when environmental concentrations of toxins are reduced and/or exposure to toxins is discontinued (Hossain et al. 2022b; Islam et al. 2019; Mrong et al. 2021; Narra et al. 2017). The water concentrations of CH gradually decreased over time and fell below the detection limit on day 9, which probably explained the recovery of the lysozyme activity after 14 days.

In this study, immune responses showed a clear two-phase pattern. On the first day after CH exposure the RBC and WBC levels and the lysozyme activity were slightly higher than in the control, suggesting an acute stress reaction where immune cells were rapidly mobilized into circulation (Dhabhar et al. 2012; Guo and Dixon 2021). During days 3 to 5, however, these parameters dropped sharply, reaching their lowest levels. This delayed suppression was likely caused by oxidative stress that damaged hematopoietic tissues and circulating leukocytes (Falfushynska et al. 2022; Moezzi et al. 2025). In addition, blood glucose remained elevated during the first 5 days, indicating a diversion of energy toward detoxification and repair processes. Such metabolic reallocation can reduce the resources available for immune activity (Barton 2002). The strong decrease in the lysozyme activity (119.2 and 105.0 U/mL in R and 2 R, respectively) indicates direct enzyme inhibition or reduced synthesis (Li et al. 2013). Notably, lysozyme activity recovered more slowly than the AChE activity and glucose levels, which may reflect fundamental differences in their underlying mechanisms. While AChE activity and glucose levels can normalize quickly once the neurotoxic stress diminishes and energy metabolism stabilizes, restoration of lysozyme requires de novo protein synthesis in immune tissues (e.g., head kidney and spleen) and recovery of leukocyte levels. These processes take longer time to rebound after toxicant-induced damage (Biller et al. 2021).

Although the immune function almost returned to the control levels by day 14, the transient suppression during days 3 to 5 could still have increased the infection risk in fish. In addition, dissolved oxygen occasionally fell to as low as 1.3 mg/L. While silver barb can tolerate short-term hypoxia, such levels are known to impose chronic stress and may act cumulatively with pesticide exposure to intensify immune suppression (Abdel-Tawwab et al. 2019; Wu 2002). Sublethal physiological changes of this kind may carry longer-term consequences for growth and reproduction that were not assessed here, but warrant investigation in future work. Compared with other insecticides, CH appears less environmentally persistent because it falls below detection levels in water within 9 days. However, its rapid onset of immunotoxic effects and potential for repeated exposures in rice–fish systems remain a concern. Similar lagged immunosuppressive effects have been documented with other pesticides (Díaz-Resendiz, Toledo-Ibarra and Girón-Pérez 2015), underscoring that even short-term exposures can have delayed and ecologically relevant impacts.

This study provides novel field-based evidence of the sublethal effects of cartap hydrochloride on silver barb under rice field conditions, yet several limitations should be acknowledged. The observation period was relatively short (14 days), only a single fish species was assessed, and water quality monitoring was limited to basic parameters. Long-term physiological, reproductive, or ecological consequences were not evaluated, and our data did not allow conclusions about environmental persistence or bioaccumulation of CH compared with other pesticides. Future research should therefore include chronic exposure studies, multi-species or community-level assessments, and experiments with pesticide mixtures that better reflect field realities, as mixtures often produce additive or synergistic effects on aquatic organisms (Cedergreen 2014; Laetz et al. 2009). In addition, studies comparing the persistence and accumulation profiles of CH with other commonly used insecticides would strengthen risk assessments (Gilliom 2007). Addressing these aspects would provide a more comprehensive understanding of risks to rice–fish systems and inform safer pesticide management strategies.

## Conclusion

This study shows that Padan 95SP (95% CH), at doses commonly used by rice farmers in the Mekong Delta, did not significantly affect the survival rate of silver barb (*Barbodes gonionotus*) fingerlings in rice fields. However, these doses caused significant effects on the brain AChE and lysozyme activity, RBC, WBC and blood glucose levels in silver barb, several days after the CH levels in water had decreased below detection levels. Brain AChE inhibition peaked at 34.0% in 2 R and 18.0% in R one day after exposure, while RBC counts dropped from 1.50 × 10⁶ to 1.03 × 10⁶ cells/mm³ during the first five days in 2 R. WBC counts declined to 93.9 × 10³ cells/mm³ in 2 R on day five compared with 130.1 × 10³ cells/mm³ in the control, and lysozyme activity fell to 105.0 U/mL in 2 R, significantly lower than the control (149.5–160.1 U/mL). This indicates a negative impact of CH on the immune defense system. During this period of time the glucose levels increased indicating an increased supply of energy to tentatively repair the damage from CH.

Despite a full recovery of the exposed fish after 14 days and only few fish mortalities, the results show that the use of CH in rice farming has negative impacts on the health of fish living in rice fields, with negative implications for the overall production of fish in the Mekong Delta. The sublethal effects of pesticides and the time lag between exposure and impact on fish are challenging to detect in the field, yet sublethal physiological changes can lead to long-term reproductivity or growth effects, which should be addressed in future work. As aquaculture and agriculture in the Mekong Delta continues to expand, it is crucial to reduce the use of pesticides like CH to ensure a stable supply of healthy food, while minimizing pollution pressure on wild and farmed fish populations. In this context, strengthening farmer outreach and training on safer pesticide practices, particularly through integrated pest management (IPM) programs, could help reduce reliance on pesticides while maintaining rice productivity. Such approaches would also protect farmer health, improve fish survival in rice–fish systems, and contribute to sustainable resource management in the region. Further research is needed to monitor sublethal effects of pesticides on aquatic organisms in rice fields, as this would provide a scientific basis for developing strategies for safer pesticide practices and improved management of aquatic resources in the Mekong Delta.

## Data Availability

No datasets were generated or analysed during the current study.

## References

[CR1] Abdel-Tawwab M, Monier MN, Hoseinifar SH, Faggio C (2019) Fish response to hypoxia stress: growth, physiological, and immunological biomarkers. Fish Physiol Biochem 45(3):997–1013. 10.1007/s10695-019-00614-9 30715663 10.1007/s10695-019-00614-9

[CR2] Amenyogbe E, Huang J, Chen G, Wang Z (2021) An overview of the pesticides’ impacts on fishes and humans. Int J Aquat Biol. 9(1):55–65. 10.22034/ijab.v9i1.972

[CR3] Anh TT, Ha NTK, Trung NQ, Huong DTT, Phuong NT (2012) Effects of quinalphos on acetylcholinesterase and growth of *Barbodes gonionotus*. CTU J Sci 22a:269–278

[CR4] Anh VT, Chiem NN, Dung DT, Sultana P (2003) Vietnam PRA Report. Understanding livelihoods dependent on inland fisheries in Bangladesh and South East Asia - Synthesis Report for FMSP Project R8118. WorldFish Center

[CR5] Asgharzadeh S, Shareghi B, Farhadian S (2024) Probing the toxic effect of chlorpyrifos as an environmental pollutant on the structure and biological activity of lysozyme under physiological conditions. Chemosphere 355:141724. 10.1016/j.chemosphere.2024.141724 38499074 10.1016/j.chemosphere.2024.141724

[CR6] Assis CRD, Bezerra RS, Luiz B, Carvalho, Jr (2011) Fish cholinesterases as biomarkers of organophosphorus and carbamate pesticides. In: Stoytcheva M (ed) Pesticides in the Modern World - Pests control and pesticides exposure and toxicity assessment. InTech, p 626

[CR7] Banerjee BD, Koner BC, Ray A (1996) Immunotoxicity of pesticides: perspectives and trends. Indian J Exp Biol 34(8):723–733 8979476

[CR8] Barton BA (2002) Stress in fishes: a diversity of responses with particular reference to changes in circulating corticosteroids. Integr Comp Biol 42(3):517–525. 10.1093/icb/42.3.517 21708747 10.1093/icb/42.3.517

[CR9] Barton BA, Iwama GK (1991) Physiological changes in fish from stress in aquaculture with emphasis on the response and effects of corticosteroids. Annu Rev Fish Dis 1:3–26. 10.1016/0959-8030(91)90019-G

[CR10] Bayne CJ, Gerwick L (2001) The acute phase response and innate immunity of fish. Dev Comp Immunol 25(8-9):725–743. 10.1016/s0145-305x(01)00033-7 11602193 10.1016/s0145-305x(01)00033-7

[CR11] Berg H, Lan THP, Da CT, Tam NT (2023) Stakeholders assessment of status and trends of ecosystem services in the Mekong Delta for improved management of multifunctional wetlands. J Environ Manage 338:117807. 10.1016/j.jenvman.2023.117807 37037143 10.1016/j.jenvman.2023.117807

[CR12] Berg H, Tam NT (2018) Decreased use of pesticides for increased yields of rice and fish-options for sustainable food production in the Mekong Delta. Sci Total Environ 619-620:319–327. 10.1016/j.scitotenv.2017.11.062 29154050 10.1016/j.scitotenv.2017.11.062

[CR13] Biller J, Polycarpo G, Schlichting Moromizato B, Sidekerskis A, Silva T, Reis I, Fierro-Castro C (2021) Lysozyme activity as an indicator of innate immunity of tilapia (*Oreochromis niloticus*) when challenged with LPS and Streptococcus agalactiae. Revista Brasileira de Zootecnia 50. 10.37496/rbz5020210053

[CR14] Boyd CE, McNevin AA (2015) Aquaculture, resource use, and the environment

[CR15] Cedergreen N (2014) Quantifying synergy: a systematic review of mixture toxicity studies within environmental toxicology. PLoS ONE 9(5):e96580. 10.1371/journal.pone.0096580 24794244 10.1371/journal.pone.0096580 PMC4008607

[CR16] Centanni M, Ricci GF, De Girolamo AM, Gentile F (2024) Modeling pesticides and ecotoxicological risk assessment in an intermittent river using SWAT. Sci Rep 14(1):6389. 10.1038/s41598-024-56991-6 38493253 10.1038/s41598-024-56991-6 PMC10944508

[CR17] Chapalamadugu S, Chaudhry GR (1992) Microbiological and biotechnological aspects of metabolism of carbamates and organophosphates. Crit Rev Biotechnol 12(5-6):357–389. 10.3109/07388559209114232 1423649 10.3109/07388559209114232

[CR18] Chen X, Yin D, Hu S, Hou Y (2004) Immunotoxicity of pentachlorophenol on macrophage immunity and IgM secretion of the crucian carp (*Carassius auratus*). Bull Environ Contam Toxicol 73(1):153–160. 10.1007/s00128-004-0407-z 15386086 10.1007/s00128-004-0407-z

[CR19] Cong NV, Danh DT, Nam TS (2021) Effects of chlorpyrifos ethyl on cholinesterase and growth of silver barb (*Barbonymus gonionotus*). CTU J Sci 13(20):2885

[CR20] Cong NV, Diem HT, Xuan TTT, Nam TS, Hang BTB (2021) Effects of Marshal 200SC on cholinesterase and growth of silver barb (*Barbonymus gonionotus*). Environ Clim Chang 57(1):90–100

[CR21] Dang T (2024) Tien Giang farmer saves freshwater specialty fish (*Barbonymus gonionotus*) that was thought to be extinct, now each fish costs millions. In: Dan Viet Newspaper. https://danviet.vn/ong-nong-dan-tien-giang-cuu-loai-ca-dac-san-nuoc-ngot-tuong-tuyet-chung-gio-gia-moi-con-tien-trieu-20240128145207563.htm. Accessed 18 January 2025

[CR22] Dhabhar FS (2009) Enhancing versus suppressive effects of stress on immune function: implications for immunoprotection and immunopathology. Neuroimmunomodulation 16(5):300–317. 10.1159/000216188 19571591 10.1159/000216188 PMC2790771

[CR23] Dhabhar FS, Malarkey WB, Neri E, McEwen BS (2012) Stress-induced redistribution of immune cells-from barracks to boulevards to battlefields: a tale of three hormones-Curt Richter Award winner. Psychoneuroendocrinology 37(9):1345–1368. 10.1016/j.psyneuen.2012.05.008 22727761 10.1016/j.psyneuen.2012.05.008 PMC3412918

[CR24] Díaz-Resendiz KJ, Toledo-Ibarra GA, Girón-Pérez MI (2015) Modulation of immune response by organophosphorus pesticides: fishes as a potential model in immunotoxicology. J Immunol Res 2015:213836. 10.1155/2015/213836 25973431 10.1155/2015/213836 PMC4417994

[CR25] Edwards P (2002) Aquaculture for poverty alleviation and food security. Aquac Asia 12(2):53–56

[CR26] Edwards P, Little DC, Yakupitiyage A (1997) A comparison of traditional and modified inland artisanal aquaculture systems. Aquac Res 28(10):777–788. 10.1046/j.1365-2109.1997.00942.x

[CR27] Ellman GL, Courtney KD, Andres Jr. V, Feather-Stone RM (1961) A new and rapid colorimetric determination of acetylcholinesterase activity. Biochem Pharmacol 7(2):88–95. 10.1016/0006-2952(61)90145-9 13726518 10.1016/0006-2952(61)90145-9

[CR28] Falfushynska H, Khatib I, Kasianchuk N, Lushchak O, Horyn O, Sokolova IM (2022) Toxic effects and mechanisms of common pesticides (Roundup and chlorpyrifos) and their mixtures in a zebrafish model (*Danio rerio*). Sci Total Environ 833:155236. 10.1016/j.scitotenv.2022.155236 35427626 10.1016/j.scitotenv.2022.155236

[CR29] Gilliom RJ (2007) Pesticides in U.S. streams and groundwater. Environ Sci Technol 41(10):3408–3414. 10.1021/es072531u 17547156 10.1021/es072531u

[CR30] Gojendro S, Chandan D, Prasanta M, Tasso T (2024) Silver barb: A promising candidate for aquaculture diversification in Meghalaya hills. Indian Farming 74(08):03–07

[CR31] Guo H, Dixon B (2021) Understanding acute stress-mediated immunity in teleost fish. Fish Shellfish Immunol Rep 2:100010. 10.1016/j.fsirep.2021.100010 36420509 10.1016/j.fsirep.2021.100010 PMC9680050

[CR32] Gupta R (2006) Toxicology of organophosphate & carbamate compounds.

[CR33] Harikrishnan R, Balasundaram C, Heo M-S (2011) Impact of plant products on innate and adaptive immune system of cultured finfish and shellfish. Aquaculture 317(1):1–15. 10.1016/j.aquaculture.2011.03.039

[CR34] Hori TS, Avilez IM, Inoue LK, Moraes G (2006) Metabolical changes induced by chronic phenol exposure in matrinxã Brycon cephalus (teleostei: characidae) juveniles. Comp Biochem Physiol C Toxicol Pharmacol 143(1):67–72. 10.1016/j.cbpc.2005.12.004 16458612 10.1016/j.cbpc.2005.12.004

[CR35] Hossain MA, Sutradhar L, Sarker TR, Saha S, Iqbal MM (2022a) Toxic effects of chlorpyrifos on the growth, hematology, and different organs histopathology of Nile tilapia, *Oreochromis niloticus*. Saudi J Biol Sci 29(7):103316. 10.1016/j.sjbs.2022.103316 36313386 10.1016/j.sjbs.2022.103316 PMC9614567

[CR36] Hossain MA, Sutradhar L, Sarker TR, Saha S, Iqbal MM (2022b) Toxic effects of chlorpyrifos on the growth, hematology, and different organs histopathology of Nile tilapia, *Oreochromis niloticus*. Saudi J Biol Sci 29(7):103316. 10.1016/j.sjbs.2022.103316 36313386 10.1016/j.sjbs.2022.103316 PMC9614567

[CR37] Hui TR, Park E, Loc HH, Tien PD (2022) Long-term hydrological alterations and the agricultural landscapes in the Mekong Delta: Insights from remote sensing and national statistics. Environ Chall 7:100454. 10.1016/j.envc.2022.100454

[CR38] Islam MA, Amin SMN, Rahman MA, Juraimi AS, Uddin MK, Brown CL, Arshad A (2022) Chronic effects of organic pesticides on the aquatic environment and human health: A review. Environ Nanotechnol Monit Manag 18:100740. 10.1016/j.enmm.2022.100740

[CR39] Islam SM, Khan MM, Moniruzzaman M, Mostakim GM, Rahman MK (2019) Recuperation patterns in fish with reference to recovery of erythrocytes in *Barbonymus gonionotus* disordered by an organophosphate. Int J Environ Sci Technol 16(11):7535–7544. 10.1007/s13762-019-02425-0

[CR40] Jern C, Wadenvik H, Mark H, Hallgren J, Jern S (1989) Haematological changes during acute mental stress. Br J Haematol 71(1):153–156. 10.1111/j.1365-2141.1989.tb06290.x 2917123 10.1111/j.1365-2141.1989.tb06290.x

[CR41] Joshi PK, Harish D, Bose M (2002) Effect of lindane and malathion exposure to certain blood parameters in a fresh water teleost fish *Clarias batrachus*. Pollut Res 21:55–57

[CR42] Khoshnood Z (2024) A review on toxic effects of pesticides in Zebrafish, Danio rerio and common carp, *Cyprinus carpio*, emphasising Atrazine herbicide. Toxicol Rep 13:101694. 10.1016/j.toxrep.2024.101694 39131695 10.1016/j.toxrep.2024.101694 PMC11314875

[CR43] Klemick H, Lichtenberg E (2008) Pesticide use and fish harvests in Vietnamese rice agroecosystems. Am J Agric Econ 90(1):1–14. 10.1111/j.1467-8276.2007.01059.x

[CR44] Kole K, Islam MR, Mrong CE et al. (2022) Toxicological effect of sumithion pesticide on the hematological parameters and its recovery pattern using probiotic in Barbonymus gonionotus. Toxicol Rep 9:230–237. 10.1016/j.toxrep.2022.02.004 35198406 10.1016/j.toxrep.2022.02.004 PMC8844800

[CR45] Laetz CA, Baldwin DH, Collier TK, Hebert V, Stark JD, Scholz NL (2009) The synergistic toxicity of pesticide mixtures: implications for risk assessment and the conservation of endangered Pacific salmon. Environ Health Perspect 117(3):348–353. 10.1289/ehp.0800096 19337507 10.1289/ehp.0800096 PMC2661902

[CR46] Li L, Cardoso JCR, Félix RC, Mateus AP, Canário AVM, Power DM (2021) Fish lysozyme gene family evolution and divergent function in early development. Dev Comp Immunol 114:103772. 10.1016/j.dci.2020.103772 32730854 10.1016/j.dci.2020.103772

[CR47] Li X, Liu L, Zhang Y, Fang Q, Li Y, Li Y (2013) Toxic effects of chlorpyrifos on lysozyme activities, the contents of complement C3 and IgM, and IgM and complement C3 expressions in common carp (*Cyprinus carpio* L.). Chemosphere 93(2):428–433. 10.1016/j.chemosphere.2013.05.023 23769463 10.1016/j.chemosphere.2013.05.023

[CR48] Majumder R (2024) Acute toxicity of chlorpyrifos to some non-target freshwater organisms: which one is more toxic—technical grade or commercial formulation? Ecotoxicology 33(10):1171–1179. 10.1007/s10646-024-02806-3 39271563 10.1007/s10646-024-02806-3

[CR49] Majumder R (2025) Effects of pesticides on fish: An overview of evolution of bioassays and cutting edge technologies. Ecol Front 45(2):286–294. 10.1016/j.ecofro.2024.11.011

[CR50] Majumder R, Kaviraj A (2017) Cypermethrin induced stress and changes in growth of freshwater fish *Oreochromis niloticus*. Int Aquat Res 9(2):117–128. 10.1007/s40071-017-0161-6

[CR51] MARD (2020) Ministry of agriculture and rural development. Circular No. 10/2020/TT-BNNPTNT. In: Development MoAaR. Ministry of Agriculture and Rural Development, Vietnam

[CR52] Mazumder SK, Debi S, Das SK, et al. (2024) Effects of extreme-ambient temperatures in silver barb (*Barbonymus gonionotus*): metabolic, hemato-biochemical responses, enzymatic activity and gill histomorphology. 16(2):292

[CR53] Mitra S, Saran RK, Srivastava S, Rensing C (2024) Pesticides in the environment: Degradation routes, pesticide transformation products and ecotoxicological considerations. Sci Total Environ 935:173026. 10.1016/j.scitotenv.2024.173026 38750741 10.1016/j.scitotenv.2024.173026

[CR54] Möck A, Peters G (2006) Lysozyme activity in rainbow trout, *Oncorhynchus mykiss* (Walbaum), stressed by handling, transport and water pollution. J Fish Biol 37(6):873–885. 10.1111/j.1095-8649.1990.tb03591.x

[CR55] Moezzi SA, Ramezani S, Rezaei K, Khoei AJ (2025) Mechanisms of pesticide toxicity in fish: insights into the ameliorative role of plant-derived compounds-a review. Aquac Nutr 2025:5328773. 10.1155/anu/5328773 40677841 10.1155/anu/5328773 PMC12267963

[CR56] Moralev SN, Rozengart. EV (2007) Comparative enzymology of Cholinesterase. International Universtity Line Inc , La Jolla, CA, USA

[CR57] Mrong CE, Islam MR, Kole K et al. (2021) Malathion-induced hematoxicity and its recovery pattern in *Barbonymus gonionotus*. J Toxicol 2021:9417380. 10.1155/2021/9417380 34970313 10.1155/2021/9417380 PMC8714397

[CR58] Mustafa SA, Al-Rudainy AJ, Salman NM (2024) Effect of environmental pollutants on fish health: An overview. Egyptian J Aquat Res 50(2):225–233. 10.1016/j.ejar.2024.02.006

[CR59] Narra MR, Rajender K, Reddy RR, Murty US, Begum G (2017) Insecticides induced stress response and recuperation in fish: Biomarkers in blood and tissues related to oxidative damage. Chemosphere 168:350–357. 10.1016/j.chemosphere.2016.10.066 27810534 10.1016/j.chemosphere.2016.10.066

[CR60] Parfitt CH (2000) Pesticide and industrial chemical residues. In: Horwitz W (ed) Official Methods of Analysis of AOAC International. vol 1—Agrochemicals; Contaminants; Drugs, 17th edn. Association of Analytical Communities International, Gaithersburg, MD, USA, p 22–23

[CR61] Park E, Loc HH, Tran DD (2024) The mekong delta environmental research guidebook. 5

[CR62] Paulson RF, Hariharan S, Little JA (2020) Stress erythropoiesis: definitions and models for its study. Exp Hematol 89:43–54.e2. 10.1016/j.exphem.2020.07.011 32750404 10.1016/j.exphem.2020.07.011 PMC7508762

[CR63] Rainboth WJ (1996) Fishes of the cambodian mekong. Food and Agriculture Organization of the United Nations, Rome. Italia

[CR64] Ram PY, Digvijay S, Singh SK, Ajay S (2003) Metabolic changes in freshwater fish Channa punctatus due to Stem-bark extract of Croton liglium. J Biol Sci 6(14):1223–1228

[CR65] Raza N (2013) Spectrophotometric determination of cartap hydrochloride in commercial samples of pesticides by iron (III) complexation. Int J Curr Pharm Res 5

[CR66] Renaud FG, Kuenzer C (2012) The mekong delta system: interdisciplinary analyses of a river delta. Springer, Dordrecht, The Netherlands . 10.1007/978-94-007-3962-8

[CR67] Saurabh S, Sahoo PK (2008) Lysozyme: an important defence molecule of fish innate immune system. Aquac Res 39(3):223–239. 10.1111/j.1365-2109.2007.01883.x

[CR68] Siwicki AK, Cossarini-Dunier M, Studnicka M, Demael A (1990) In vivo effect of the organophosphorus insecticide trichlorphon on immune response of carp (Cyprinus carpio). II. Effect of high doses of trichlorphon on nonspecific immune response. Ecotoxicol Environ Saf 19(1):99–105. 10.1016/0147-6513(90)90084-i 2311567 10.1016/0147-6513(90)90084-i

[CR69] Sogorb MA, Vilanova E (2002) Enzymes involved in the detoxification of organophosphorus, carbamate and pyrethroid insecticides through hydrolysis. Toxicol Lett 128(1):215–228. 10.1016/S0378-4274(01)00543-4 11869832 10.1016/s0378-4274(01)00543-4

[CR70] Stadlinger N, Berg H, Van den Brink PJ, Tam NT, Gunnarsson JS (2018) Comparison of predicted aquatic risks of pesticides used under different rice-farming strategies in the Mekong Delta, Vietnam. Environ Sci Pollut Res Int 25(14):13322–13334. 10.1007/s11356-016-7991-4 27854060 10.1007/s11356-016-7991-4 PMC5978820

[CR71] Stenersen J (2004) Chemical pesticides: mode of action and toxicity. CRC Press, Boca Raton, London

[CR72] Stolen JS (1995) Techniques in fish immunology: immunological and pathological techniques of aquatic invertebrates. SOS Publications, Fair Haven, NJ, USA

[CR73] Sule RO, Condon L, Gomes AV (2022) A common feature of pesticides: oxidative stress-The role of oxidative stress in pesticide-induced toxicity. Oxid Med Cell Longev 2022:5563759. 10.1155/2022/5563759 35096268 10.1155/2022/5563759 PMC8791758

[CR74] Tam NT, Berg H, Laureus J, Cong NV, Tedengren M (2016) Effects of Sequential Applications of Bassa 50EC (Fenobucarb) and Vitashield 40EC (Chlorpyrifos ethyl) on Acetylcholinesterase Activity in Climbing Perch (*Anabas testudineus*) Cultured in Rice Fields in the Mekong Delta, Vietnam. Bull Environ Contam Toxicol 97(1):98–104. 10.1007/s00128-016-1796-5 27075585 10.1007/s00128-016-1796-5

[CR75] Tien C-J, Lin M-C, Chiu W-H, Chen CS (2013) Biodegradation of carbamate pesticides by natural river biofilms in different seasons and their effects on biofilm community structure. Environ Pollut 179:95–104. 10.1016/j.envpol.2013.04.009 23665845 10.1016/j.envpol.2013.04.009

[CR76] Toan PV, Sebesvari Z, Blasing M, Rosendahl I, Renaud FG (2013) Pesticide management and their residues in sediments and surface and drinking water in the Mekong Delta, Vietnam. Sci Total Environ 452:28–39. 10.1016/j.scitotenv.2013.02.026 23500396 10.1016/j.scitotenv.2013.02.026

[CR77] Tomašević A, Mijin D, Marinkovic A, Cvijetić I, Gasic S (2019) Photocatalytic degradation of carbamate insecticides: Effect of different parameters. Pesticidi i fitomedicina 34:193–200. 10.2298/PIF1904193T

[CR78] Ullah M, Yousafzai AM, Muhammad I et al. (2022) Effect of cypermethrin on blood hematology and biochemical parameters in fresh water fish *Ctenopharyngodon idella* (Grass Carp). Cell Mol Biol 68(10):15–20. 10.14715/cmb/2022.68.10.3 10.14715/cmb/2022.68.10.3 PMC10155271 37114278

[CR79] van der Oost R, Beyer J, Vermeulen NPE (2003) Fish bioaccumulation and biomarkers in environmental risk assessment: a review. Environ Toxicol Pharmacol 13(2):57–149. 10.1016/S1382-6689(02)00126-6 21782649 10.1016/s1382-6689(02)00126-6

[CR80] Viet D (2015) When visiting Cần Thơ, don’t miss the chance to savor “chả cá mè vinh (silver barb) kho mẳn”. In: Can Tho City Electronic Information Portal. https://www.cantho.gov.vn/wps/portal/home/du-khach/chi-tiet/am-thuc/mon-ngon-can-tho/den+can+tho+nho+an+cha+ca+me+vinh+kho+man?WCM_Page.Menu_TinTucKhac=3#:~:text=C%C3%A1%20m%C3%A8%20vinh%20th%E1%BB%8Bt%20ng%E1%BB%8Dt,c%C3%A1%20m%C3%A8%20vinh%20kho%20m%E1%BA%B3n

[CR81] Wendelaar Bonga SE (1997) The stress response in fish. Physiol Rev 77(3):591–625. 10.1152/physrev.1997.77.3.591 9234959 10.1152/physrev.1997.77.3.591

[CR82] Winkaler EU, Santos TRM, Machado-Neto JG, Martinez CBR (2007) Acute lethal and sublethal effects of neem leaf extract on the neotropical freshwater fish Prochilodus lineatus. Comp Biochem Physiol C Toxicol Pharmacol 145(2):236–244. 10.1016/j.cbpc.2006.12.009 17251062 10.1016/j.cbpc.2006.12.009

[CR83] Wu RSS (2002) Hypoxia: from molecular responses to ecosystem responses. Mar Pollut Bull 45(1):35–45. 10.1016/S0025-326X(02)00061-9 12398365 10.1016/s0025-326x(02)00061-9

[CR84] Yang C, Lim W, Song G (2021) Immunotoxicological effects of insecticides in exposed fishes. Comp Biochem Physiol C Toxicol Pharmacol 247:109064. 10.1016/j.cbpc.2021.109064 33905824 10.1016/j.cbpc.2021.109064

[CR85] Zahran E, Risha E, Awadin W, Palić D (2018) Acute exposure to chlorpyrifos induces reversible changes in health parameters of Nile tilapia (*Oreochromis niloticus*). Aquat Toxicol 197:47–59. 10.1016/j.aquatox.2018.02.001 29433082 10.1016/j.aquatox.2018.02.001

